# Daily Oral Pre-exposure Prophylaxis (PrEP) Continuation Among Women from Durban, South Africa, Who Initiated PrEP as Standard of Care for HIV Prevention in a Clinical Trial

**DOI:** 10.1007/s10461-022-03592-x

**Published:** 2022-02-05

**Authors:** Ivana Beesham, Dvora L. Joseph Davey, Mags Beksinska, Shannon Bosman, Jenni Smit, Leila E. Mansoor

**Affiliations:** 1 MatCH Research Unit (MRU), Department of Obstetrics and Gynecology, Faculty of Health Sciences, University of the Witwatersrand, Durban, South Africa; 2 Division of Epidemiology and Biostatistics, School of Public Health and Family Medicine, University of Cape Town, Cape Town, South Africa; 3 Department of Epidemiology, Fielding School of Public Health, University of California Los Angeles, Los Angeles, CA USA; 4 Centre for the AIDS Programme of Research in South Africa (CAPRISA), University of KwaZulu-Natal, Durban, South Africa

**Keywords:** Oral pre-exposure prophylaxis (PrEP), PrEP continuation, HIV prevention, Women, Clinical trials, South Africa

## Abstract

HIV incidence among women in Eastern and Southern Africa remains unacceptably high, highlighting the need for effective HIV prevention options, including pre-exposure prophylaxis (PrEP). The Evidence for Contraceptive Options and HIV Outcomes trial offered daily oral PrEP to participants during the latter part of the clinical trial as an additional HIV prevention choice. We explored daily oral PrEP continuation at trial exit among women enrolled from Durban, South Africa who initiated oral PrEP at the trial site. Of the 132 women initiating oral PrEP, 87% reported continuation of oral PrEP at month 1, 80% at month 3, and 75% continued using oral PrEP at their final trial visit and were referred to off-site facilities for ongoing oral PrEP access. The median duration of oral PrEP use in trial participants who used oral PrEP was 91 days (IQR 87 to 142 days). Women who disclosed their oral PrEP use to someone had increased odds of continuing oral PrEP at trial exit. Women who reported > 1 sex partner and those who felt they would probably or definitely get infected with HIV had reduced odds of continuing oral PrEP at trial exit. Of those discontinuing oral PrEP (n = 32), > 50% discontinued within the first month, and the most common reason for discontinuation was reporting side effects. The high rates of oral PrEP continuation in our study are encouraging and our findings can be utilized by other clinical trials providing oral PrEP as standard of care for HIV prevention and by oral PrEP implementation programmes.

## Introduction

Over the last decade, substantial progress has been made in reducing HIV infections in Eastern and Southern Africa, with a 38% decline in new infections since 2010, however, adolescent girls and young women (AGYW) continue to account for over one-quarter of incident HIV infections in the region [1]. In South Africa, in 2019, the HIV prevalence among women aged 15 to 49 years was 25%, and 120,000 women aged 15 years and older became infected with HIV [2]. The main mode of transmission of HIV in this setting is via heterosexual sex [3]. Daily oral pre-exposure prophylaxis (PrEP) is a single pill consisting of tenofovir disoproxil fumarate and emtricitabine that reduces the risk of HIV transmission during sex by about 99% when taken daily [4]. In 2015, the World Health Organization (WHO) included daily oral PrEP as an additional prevention choice for those at substantial risk of HIV infection as part of combination prevention [5]. Globally, almost a million people had initiated oral PrEP by the end of 2020, with over 100,000 people in South Africa [6].

Despite progress in the uptake of oral PrEP, studies have shown that many users discontinue oral PrEP within 1 to 3 months, and continuation rates have been low [7–10]. A systematic meta-analysis that included clinical trials, demonstration projects and real-world settings on oral PrEP continuation found that on average 65% continued oral PrEP at month 1 [7]. Other studies in sub-Saharan Africa found even lower rates of oral PrEP continuation, for example, in the DREAMS study among AGYW in Kenya, 37% of women continued oral PrEP at 3 months [8]; in the PRIYA programme in Kenya, 41% continued at month 1, and 24% at month 3 [9]; and in the POWER study conducted among AGYW in Kenya and South Africa, 30% continued at month 1 and 21% through 6 months [10]. Reasons for oral PrEP discontinuation have included side effects (perceived and/or experienced), difficulties with a daily pill regimen, stigma, a lack of social support, disapproval from family and sexual partners, a reduction in HIV risk and challenges in accessing oral PrEP [11–13].

In the context of clinical trials, current global ethical guidelines advise that researchers and trial sponsors in HIV prevention studies should, at minimum, provide the recommended package of HIV prevention methods as stipulated by WHO, which includes oral PrEP for those at substantial risk of HIV [14]. In addition, in 2017, the South African Medical Research Council (SAMRC) recommended that oral PrEP be provided as HIV prevention standard of care at clinical trial sites conducting HIV prevention research [15]. The aim of this study was to describe oral PrEP continuation, the timing and reasons for oral PrEP discontinuation, and factors associated with oral PrEP continuation, among women from Durban, South Africa who were enrolled in the Evidence for Contraceptive Options and HIV Outcomes (ECHO) trial. The ECHO trial was a multi-centre randomized clinical trial conducted between 2015 and 2018, at 12 sites in four African countries, and the primary outcome was HIV incidence among women randomized to one of three contraceptives [16]. The ECHO trial was one of the first HIV endpoint-driven clinical trials in Africa to incorporate oral PrEP as part of the HIV prevention package provided to study participants. During the latter 8 months of the ECHO trial, oral PrEP was provided onsite to women as part of a comprehensive HIV prevention package. Given that HIV endpoint-driven clinical trials enrol persons at high risk for HIV acquisition and many oral PrEP users discontinue oral PrEP early, data on oral PrEP continuation when oral PrEP is provided as standard of care in clinical trials is important for current and future HIV endpoint-driven clinical trials.

## Methods

### Study Population and Procedures

HIV-negative, sexually active women, aged 18 to 35 years, desiring contraception, and willing to receive any of the three study contraceptives (intramuscularly injected depot medroxyprogesterone acetate, the copper intrauterine device or levonorgestrel implant) with no contraindications to any of the methods were enrolled into the ECHO trial at the Durban, South Africa trial site. Follow-up was quarterly for 12–18 months. The ECHO trial was conducted from December 2015 to October 2018 [16]. We conducted an ancillary study nested within the ECHO trial to collect additional data on oral PrEP use among women who initiated oral PrEP onsite at the Durban trial site and the primary results of this ancillary study have been published [17]. For this analysis, we describe oral PrEP continuation and factors associated with continuation at study exit among women who initiated oral PrEP onsite at the ECHO trial site in Durban, South Africa.

### Onsite Oral PrEP Provision During the ECHO Trial

The integration of oral PrEP within the ECHO trial has been previously described [18]. Briefly, oral PrEP was provided onsite on a voluntary basis at the Durban trial site from March till October 2018 as part of a comprehensive HIV prevention package. Some women who had been enrolled earlier in the ECHO trial had been exited prior to oral PrEP being made available onsite. Women were eligible for onsite oral PrEP provision if they attended a study visit from March 2018, were HIV negative, not pregnant or breastfeeding (as per local guidelines at the time) and had at least 1 month of follow-up remaining in the trial. Staff were trained at the trial site using components that were adapted from the South African standardized PrEP training package, and the South African National Department of Health (NDoH) PrEP Guidelines [18]. Field-tested material developed by the Southern African HIV Clinicians Society, the NDoH, and an Optimizing Prevention Technology Introduction on Schedule were also used [18]. The training included provider sensitization, as well as the importance of participants exploring their own risk profile and risk reduction options, and adherence to and effective use of oral PrEP.

At the Durban site, during HIV risk-reduction counselling at each follow-up visit, counsellors discussed HIV prevention options with women, including daily oral PrEP. Those who were interested in initiating oral PrEP were referred to study clinicians for same day initiation. Women who initiated oral PrEP were seen 1 month after initiation and thereafter every 2–3 months. Oral PrEP was dispensed onsite by trial clinicians. A 1-month supply of oral PrEP was given at initiation, and thereafter, refills were given approximately every 3 months. Women were counselled on oral PrEP adherence at initiation and follow-up visits by trial staff. Where possible PrEP visits were integrated with the main ECHO trial study visits. At the final study visit, women who desired to continue using oral PrEP were given a 3-month supply and referred to off-site facilities for oral PrEP refills, such as local demonstration projects and public sector clinics providing oral PrEP. Women were given a referral letter and details about the off-site oral PrEP facility including the physical address. In addition, where feasible, women were provided with transport from the research site to the off-site facility for the first visit if they felt they would be unable to locate the facility independently.

Women could choose to discontinue oral PrEP at any time point. Women who seroconverted discontinued oral PrEP and were referred for HIV care. HIV resistance testing was conducted among oral PrEP users that seroconverted and results were provided to women and their healthcare providers.

### Oral PrEP Continuation

For this analysis, oral PrEP continuation was defined as women who had initiated oral PrEP onsite at any time during the course of the trial, prior to the final trial visit, who continued using oral PrEP at their final trial visit and were referred for off-site oral PrEP access at the final visit. We also report on oral PrEP continuation at month 1 and month 3 post PrEP initiation. The duration of oral PrEP use was limited by follow-up time remaining in the trial for each participant from the date of oral PrEP initiation.

### Oral PrEP Adherence

Self-reported adherence to oral PrEP was collected over the prior 30 days among women who reported ongoing oral PrEP use (had used oral PrEP within the prior 30 days) at the time of the questionnaire. As previously reported, 37% of women reported no missed doses in the prior 30 days [17].

### Data Collection

Data that were collected during the main ECHO trial such as participant demographics, sexual risk behaviours and dates of oral PrEP use were recorded using case report forms (CRFs). At each study visit, women were asked a question on whether they had used oral PrEP since the previous visit, and if they had taken oral PrEP, dates of use were recorded on study CRFs by clinicians. If oral PrEP was discontinued, the date of discontinuation was recorded on CRFs. For the ancillary study, among women who had initiated oral PrEP on-site, an additional interviewer administered questionnaire was administered. Data collected for the ancillary study included reasons for discontinuing oral PrEP, oral PrEP disclosure, HIV risk-perception and timing of oral PrEP discontinuation. Data for the ancillary study were entered onto the REDCap® electronic data capture tools hosted at the University of the Witwatersrand [19].

### Sexually Transmitted Infection (STI) Testing

STI testing was conducted at baseline via provider collected swabs and nucleic acid amplification testing for *Chlamydia trachomatis* (CT) and *Neisseria gonorrhoeae* (NG). Women who had clinical signs of STIs and/or diagnoses of STIs based on laboratory testing were treated.

### Data Analysis

We evaluated baseline factors at enrolment into the ECHO trial (including age, education, relationship status, STIs, and partner HIV status), sexual behaviours at oral PrEP initiation (including the number of sexual partners and condom use), and HIV risk perception and disclosure of oral PrEP use approximately 3 months after oral PrEP initiation, using descriptive analyses. We report on frequencies and proportions for categorical variables. Continuous variables such as age, number of sex acts, and number of partners were converted into categorical variables.

We constructed univariate logistical regression models to evaluate factors associated with oral PrEP continuation at study exit. Factors associated with oral PrEP continuation at *p* < 0.05 were included in the multivariate analysis in which we adjusted for a priori confounders (age, relationship status). The model results are presented as crude and adjusted odds ratios (OR and aOR) with 95% confidence intervals (CIs). All statistical analyses were conducted with STATA v14.1 [20].

### Ethics

Women signed written informed consent to participate in the ECHO trial, and additional informed consent was obtained to participate in the ancillary study. The ECHO trial was approved by the University of the Witwatersrand Human Research Ethics Committee (Wits HREC) and the FHI360 ethics review board. The ancillary study was approved by Wits HREC (Reference 141112).

## Results

In total, 138 women who were enrolled in the ECHO trial initiated oral PrEP onsite at the Durban trial site. Of these, 132 (96%) consented to participate in the ancillary study. Among women enrolled in the ancillary study, the median age was 23 years (range 18–35 years) and almost two-thirds were ≤ 24 years (62%) (Table 1). Most women were unmarried (97%) and not living with their partner (92%). A quarter of the women had CT detected at baseline, and 2% had NG*.* At oral PrEP initiation, 7% of women reported having two sexual partners, and two-thirds (67%) reported inconsistent or non-use of condoms over the prior 3 months.

**Table 1:** Participant characteristics at baseline and oral PrEP initiation visit (n = 132)

Characteristic	n (%)
*Baseline characteristics*	
Age	
18–24	82 (62)
25–30	41 (31)
31–35	9 (7)
Education	
Secondary (incomplete)	49 (37)
Secondary (complete)	46 (35)
Post-secondary	37 (28)
Marital status	
Married	3 (2)
Never married	128 (97)
Separated	1 (1)
Lives with partner	
Yes	9 (7)
No	122 (92)
No partner	1 (1)
Earns an income	
Yes	22 (17)
No	110 (83)
STI diagnosis	
Chlamydia trachomatis	33 (25)
Neisseria gonorrhoea	3 (2)
Partner HIV status	
Positive	0 (0)
Negative	112 (85)
Unknown	20 (15)
*Characteristics at PrEP initiation visit*	
Number of sexual partners in last 3 months	
0	1 (1)
1	122 (92)
2	9 (7)
No of sexual acts in the last 3 months^a^	
0	8 (6)
1–3	42 (32)
4–6	36 (27)
7–9	14 (11)
10–12	14 (11)
13–20	8 (6)
> 20	9 (7)
Condom frequency during the last 3 months	
Never	23 (17)
Rarely	2 (2)
Sometimes	53 (40)
Often	11 (8)
Always	34 (26)
No sex partner/did not have sex	9 (7)

^a^Applies to participants who had a sexual partner in the last 3 months (n = 131)

**Fig. 1:**
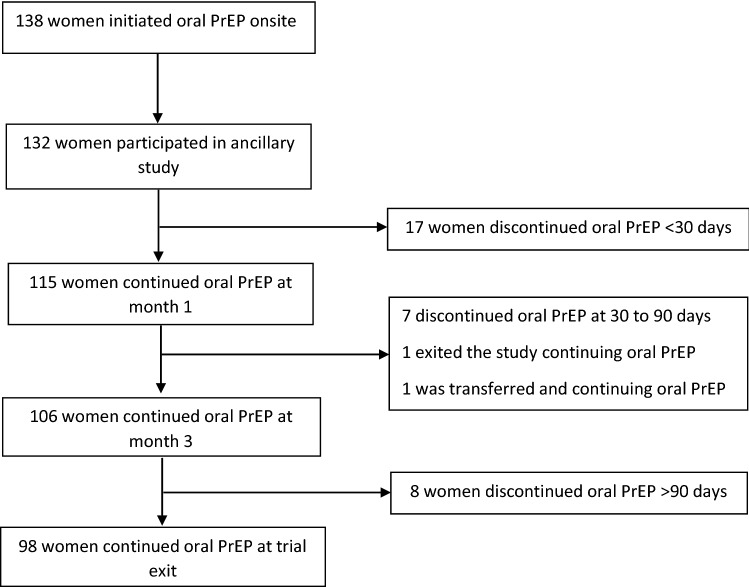
Oral PrEP continuation during study follow-up

Of the 132 women who initiated oral PrEP in the trial, 87% (n = 115) continued using oral PrEP at month 1, and 80% (n = 106) at month 3 (Fig. 1). The median duration of oral PrEP use among all users during follow-up was 91 days (IQR 87 to 142 days). Overall, 75% of women (n = 99) had elected to continue using oral PrEP at their final trial visit. Among the 32 women who had discontinued oral PrEP, three-quarters (n = 24, 75%) had discontinued within the first 3 months. The most frequent reason for discontinuing oral PrEP was reporting side effects (n = 12, 38%), and half of the women who discontinued due to experiencing side effects, had done so within the first 30 days of starting oral PrEP (Table 2). Other reasons for discontinuing included being influenced by a partner or family member to stop (16%) and forgetting to take oral PrEP (13%). Fewer than 10% of women discontinued oral PrEP because they no longer felt at risk of acquiring HIV.

**Table 2:** Timing and reasons for oral PrEP discontinuation up to the final trial visit (n = 32)

Reasons for oral PrEP discontinuation^a^	n (%)
*Discontinued oral PrEP < 30 days*	
Side effects for example, nausea, vomiting	6 (19)
Influenced by the partner/family to stop using oral PrEP	4 (13)
Afraid of potential side effects related to oral PrEP use	2 (6)
Was unable to return to study site for a refill	2 (6)
Decided to use condoms instead	1 (3)
Forgetful to take oral PrEP	1 (3)
Was going away from study site for more than a month and felt she would run out of tablets	1 (3)
Stopped due to religious reasons	1 (3)
*Discontinued oral PrEP at 30 to 90 days*	
Side effects for example headaches, vomiting	5 (16)
Forgetful to take oral PrEP	1 (3)
No longer at risk (no partner)	1 (3)
*Discontinued oral PrEP > 90 days*	
Forgetful to take oral PrEP	2 (6)
No longer at risk (no partner/partner is going away for a few months)	2 (6)
Does not have time to attend clinic for oral PrEP refills	1 (3)
Influenced by partner/family to stop using oral PrEP	1 (3)
Does not want to take a daily pill	1 (3)
Does not want a high pill burden (on oral tablets for a medical condition)	1 (3)
Side effects (fatigue)	1 (3)

^a^Multiple reasons allowed

Among women who disclosed oral PrEP use to family members, partners and/or friends (n = 118), 79% (93 of 118) continued using oral PrEP at the final visit. Women who disclosed oral PrEP use had almost five times the odds of continuing using oral PrEP at the final study visit compared to those who did not disclose that they were taking oral PrEP (aOR 4.98; 95% CI 1.45, 17.13; p = 0.01), adjusted for age and relationship status (Table 3). Women who had more than one sex partner (n = 9, 7%) had lower odds of continuing oral PrEP compared to those who had one sex partner (aOR 0.24; 95% CI 0.06, 0.97; p = 0.05), adjusted for age and relationship status. Among women who reported that they felt they would probably or definitely get infected with HIV (n = 30), over half (57%) continued using oral PrEP at the final trial visit. Women who felt they would probably or definitely get infected with HIV had lower odds of continuing oral PrEP compared to women who felt they would definitely or probably not get infected with HIV (aOR 0.21; 95% CI 0.08, 0.55; p < 0.01), adjusted for age and relationship status.

**Table 3:** Factors associated with self-reported oral PrEP continuation at the final study visit (median of 3 months) among 131 women

Characteristic	Total n (%)	Oral PrEP continuation at the final trial visit (median = 91 days) n (%)		Odds ratio (OR) (95% confidence interval [CI])	p value	Adjusted OR (95% CI)^a^	p value
		Yes	No				
Age > 24 years	50 (38)	40 (40)	10 (31)	1.49 (0.64, 3.48)	0.36		
Age ≤ 24 years (ref)	81 (62)	59 (60)	22 (69)				
Education (secondary level or higher)	82 (63)	62 (63)	20 (63)	1.01 (0.44, 2.29)	0.99		
Education (secondary level—incomplete) (ref)	49 (37)	37 (37)	12 (38)				
Relationship status					0.35		
Lives with partner	9 (7)	8 (8)	1 (3)	2.76 (0.33, 22.92)			
Does not live with partner (ref)	121 (93)	90 (92)	31 (97)				
Earns an income	22 (17)	18 (18)	4 (13)	1.56 (0.48, 4.99)	0.46		
Does not earn an income (ref)	109 (83)	81 (82)	28 (88)				
STI diagnosis at enrolment							
Chlamydia trachomatis detected	32 (24)	22 (22)	10 (31)	0.63 (0.26, 1.52)	0.30		
Chlamydia trachomatis not detected (ref)	99 (76)	77 (78)	22 (69)				
Neisseria gonorrhoea detected	3 (2)	3 (3)	0 (0)	–	–		
Neisseria gonorrhoea not detected (ref)	128 (98)	96 (97)	32 (100)				
Unknown partner HIV status	20 (15)	14 (14)	6 (19)	0.71 (0.25, 2.04)	0.53		
Partner is HIV-negative (ref)	111 (85)	85 (86)	26 (81)				
More than 1 sex partner	9 (7)	4 (4)	5 (16)	0.23 (0.06, 0.91)	**0.04**	0.24 (0.06, 0.97)	**0.05**
Has 0/1 sex partner (ref)	122 (93)	95 (96)	27 (84)				
> 12 sex acts in the last 3 months	17 (14)	13 (14)	4 (14)	1.02 (0.30, 3.40)	0.97		
≤ 12 sex acts in last 3 months (ref)	105 (86)	80 (86)	25 (86)				
Any unprotected sex in last 3 months	88 (72)	67 (72)	21 (72)	0.98 (0.39, 2.49)	0.97		
No unprotected sex (always used condoms) (ref)	34 (28)	26 (28)	8 (28)				
HIV risk perception						0.21 (0.08, 0.55)	** < 0.01**
I will probably/definitely get infected with HIV	30 (25)	17 (18)	13 (48)	0.24 (0.10, 0.60)	** < 0.01**		
I will probably/definitely not get infected with HIV (ref)	90 (75)	76 (82)	14 (52)				
Disclosed oral PrEP use	118 (90)	93 (94)	25 (78)	4.34 (1.34, 14.07)	**0.01**	4.98 (1.45, 17.13)	**0.01**
Did not disclose oral PrEP use (ref)	13 (10)	6 (6)	7 (22)				

Bold values indicate *p* ≤ 0.05

^a^Factors associated with oral PrEP continuation at *p* < 0.05 are included in the multivariate analysis in which we adjust for a priori confounders (age (continuous) and relationship status)

Less than half of the women who did not disclose oral PrEP use (6 of 13, 46%) continued oral PrEP at the final trial visit. Among women who reported that they felt they would probably or definitely not get infected with HIV (n = 90), 76 (84%) continued oral PrEP at the final study visit.

One woman who initiated oral PrEP seroconverted. She reported discontinuing oral PrEP approximately 2 months prior to seroconversion.

## Discussion

In this ancillary PrEP study, where daily oral PrEP was offered onsite as part of HIV prevention standard of care during the latter part of the ECHO trial, we found that oral PrEP continuation during the trial and at the final trial visit, among women enrolled at the Durban trial site was high, with 87% of women continuing oral PrEP at month 1, 80% at month 3, and 75% electing to continue using oral PrEP at trial exit. Of the 25% of women who reported discontinuing oral PrEP, three-quarters had discontinued within the first 3 months, and side effects were the most frequent reason for oral PrEP discontinuation. Women who disclosed oral PrEP use continued oral PrEP more than those who did not disclose, whilst women who reported having more than one sexual partner and those who felt that they had a high risk of HIV infection had reduced odds of continuing oral PrEP at the final study visit. As reported previously, 43% of women who were eligible for oral PrEP when it became available, had initiated oral PrEP onsite [17].

We found that the proportion of women continuing oral PrEP at month 1 and month 3 in our study was higher than in some studies conducted among AGYW in South Africa and Kenya [8, 10] such as the DREAMS study in Kenya, where only 37% of AGYW who initiated oral PrEP, persisted with PrEP use at month 3 [8]. Similarly, in a real-world implementation programme in Kenya (PrIYA), where oral PrEP was integrated into family planning clinics in Kenya, less than half of the women who initiated oral PrEP had returned for a PrEP refill at month 1 [9]. A recent study conducted in South Africa among pregnant women who initiated oral PrEP during pregnancy found higher rates of oral PrEP continuation where three-quarters of women continued at month 1 and 62% at months 3 [21]. In this study among pregnant women, factors associated with an increased odds of continuing oral PrEP at month 3 included having a partner who was living with HIV or of unknown serostatus, and reporting intimate partner violence [21]. In the HPTN 082 study which enrolled AGYW from Zimbabwe and South Africa and had good retention (~ 90% at month 3), 84% of women had detectable levels of tenofovir-diphosphate at month 3 [22]. Oral PrEP continuation in this study might have been facilitated by weekly 2-way text messaging, adherence counselling, and voluntary participation in monthly adherence clubs.

The high rates of oral PrEP continuation reported by participants in our study could be attributed to several factors. First, oral PrEP was offered and provided as part of a comprehensive HIV prevention package in the ECHO trial and was integrated with contraceptive provision and care. Where possible, PrEP study visits were aligned with regular study follow-up visits. Second, oral PrEP was provided onsite, at no cost to participants, and this removed several barriers to oral PrEP access. Third, oral PrEP use was facilitated by trial staff. Staff received training on oral PrEP provision, including the importance of participants exploring their own risk profile and risk reduction options, and adherence to and effective use of oral PrEP. Oral PrEP information and materials were readily available to study participants, and separate workshops and presentations on oral PrEP were conducted with study community advisory boards [18].

Reasons for oral PrEP discontinuation in our study are consistent with other published studies [9, 12, 22]. An important consideration when assessing oral PrEP use is the use of oral PrEP during periods of risk and stopping oral PrEP when no longer at risk, and recently, there has been much discussion on “prevention-effective” adherence [23, 24]. In our study, we found that some women discontinued using oral PrEP as they no longer felt at risk for acquiring HIV. Among women who discontinued oral PrEP, almost 40% had done so due to side effects and this highlights the need for ongoing counselling and effective management of side effects among oral PrEP users, especially during the first few weeks of oral PrEP use. As oral PrEP was offered relatively late into the trial, overlapping side effects from contraceptives and oral PrEP were less of a concern, as contraceptive use had been established in most women.

In our study, most women who initiated oral PrEP (90%) had disclosed oral PrEP use to family members, partners and/or friends, and those who had disclosed oral PrEP use had almost five times the odds of continuing oral PrEP at trial exit compared to women who did not disclose. However, even among those who discontinued oral PrEP (n = 32), almost 80% had disclosed oral PrEP use. A study conducted among AGYW in South Africa found that disclosure can be a facilitator or barrier to oral PrEP use—supportive disclosures could provide instrumental adherence support such as pill reminders, however, unsupportive disclosures could undermine oral PrEP adherence and subject women to stigma, and in some cases, result in oral PrEP discontinuation [25]. An additional consideration is who a person discloses to, and the study among AGYW found that partners and parents provided the most support to participants who disclosed oral PrEP use [25]. Stigma was also found to be a barrier to oral PrEP continuation among pregnant women in South Africa [26]. We found that women who felt they would probably or definitely get infected with HIV had reduced odds of continuing oral PrEP at the final study visit. This might be related to the timing of the questionnaire, as it was administered approximately 3 months after oral PrEP initiation, and women might have felt less at risk because of using daily oral PrEP. Alternatively, it is possible that women who felt at increased risk for HIV acquisition experienced additional challenges and barriers to oral PrEP continuation such as partner-related issues (i.e., difficulties with disclosure of oral PrEP use and issues relating to trust and/or fidelity in the relationship) or stigma (fear that others may think that oral PrEP is treatment for HIV) [26]. Surprisingly, women who had more than one sex partner had reduced odds of continuing oral PrEP, and this is an area that requires further exploration. However, this finding was limited to a small number of women who reported more than one sex partner (n = 9).

One of the strengths of this study was that all women who had initiated oral PrEP onsite remained in the ECHO trial until oral PrEP discontinuation, transfer to another study site or trial exit, therefore, we do not have missing data on women who did not return for an oral PrEP refill. Limitations of this study include that oral PrEP was introduced relatively late during the ECHO trial, therefore, the duration of oral PrEP use and continuation rates were limited by study follow-up time. Continuation rates beyond this time would have been useful to understand longer term oral PrEP use, including stopping and restarting oral PrEP. We enrolled women from one study site only and our sample size was small, hence, our findings may not be generalizable to other populations and settings. Furthermore, this was a clinical trial population who were research literate and being reimbursed for study participation, hence our findings might not be generalizable to oral PrEP users in real-world settings such as oral PrEP demonstration and implementation projects. In addition, we reported on oral PrEP continuation via participant self-report, and this might not correlate with adherence to oral PrEP as we did not conduct any objective measures of adherence such as measuring tenofovir drug levels. Finally, in-depth interviews would have been useful to gain deeper insights into reasons for oral PrEP continuation among clinical trial participants.

## Conclusions

The high rates of oral PrEP continuation among women participating in the ECHO trial in Durban, South Africa who initiated oral PrEP are encouraging. Three-quarters of women who discontinued oral PrEP had discontinued within 3 months of initiating oral PrEP. This highlights the importance of providing additional support, counselling and side effect management especially during the first few months of oral PrEP use. Disclosure of oral PrEP use was positively associated with oral PrEP continuation, however overall disclosure rates were high in our study population, and this is an area that requires further exploration. Additional research exploring HIV risk perception both at oral PrEP initiation and subsequently, is needed to better understand oral PrEP use. Our findings can be used by other clinical trials providing oral PrEP as standard of care for HIV prevention, and importantly, may inform oral PrEP programmes as oral PrEP is being scaled up in South Africa, and other countries in sub-Saharan Africa.

## Data Availability

Access to data from the ECHO Study may be requested through submission of a research concept to icrc@uw.edu. The concept must include the research question, data requested, analytic methods, and steps taken to ensure ethical use of the data. Access will be granted if the concept is evaluated to have scientific merit and if sufficient data protections are in place. As of the time of publication, data access applications are in process with the governing institutional review boards of the ECHO Study to make de-identified data publicly available.
